# Comparison of goalscoring patterns between the 2018 and 2022 FIFA World Cups

**DOI:** 10.3389/fspor.2024.1394621

**Published:** 2024-05-22

**Authors:** Olivier Degrenne, Christopher Carling

**Affiliations:** ^1^ Val de Marne, Université Paris-Est Créteil Val de Marne, Creteil, France; ^2^ French Football Federation Research Center, Clairefontaine National Football Centre, Clairefontaine-en-Yvelines, France; ^3^ Laboratory Sport, Expertise, and Performance (EA 7370), French Institute of Sport (INSEP), Paris, France

**Keywords:** football, match analysis, performance, goals, World Cup

## Abstract

The main aim of this study was to compare goal scoring patterns between the two most recent FIFA World Cup^TM^ (WC) tournaments: Russia 2018 and Qatar 2022. Match performance data were extracted using the ProVision database (StatsPerform, Chicago, USA). Variables used to analyse goals (not including penalty shootout goals) included the match period in which these were scored, the type of action and different types of play, body part used, defensive pressure, possession duration and expected goals (xG) values. A Chi-square test was used to compare qualitative variables across competitions while quantitative variables were compared using a nonparametric Mann–Whitney *U*-test for independent samples. Altogether, 169 goals were scored in WC 2018 vs. 172 goals in WC 2022. No differences occurred across competitions for the period in which goals were scored. In both tournaments the majority was scored in the second-half and towards the end of play. Significant differences were observed for the type of action, type of play and body part used prior to scoring a goal (*p* < .05). These differences can be explained by the record number of own goals and goals scored from set-pieces in 2018 and an increase in goals scored from open-play (from positional attacks notably) in 2022. Analysis of open-play situations showed that teams recorded a higher xG value and converted more of their chances (xG = 122.8 vs. 128 goals, xG difference = +5.2) in 2022 thus were more efficient compared with 2018. In sum, goal scoring patterns largely differed between the two most recent World Cup tournaments.

## Introduction

Over the past two decades, performance analysis has become an integral part of the coaching process, coupled with an increase in published research on the topic (1). The analysis of match-play performance provides objective information that allows coaches to identify collective and individual strengths and weaknesses, and subsequently provide feedback to ultimately enhance technical behaviour and tactical decision-making (2). The most important single factor that determines results in football play is evidently the number of goals scored (or conversely, conceded). Strongly linked to this is the ability of teams to create scoring chances and achieve high levels of efficiency in converting these actions (e.g., frequency and proportion of shots on target). Indeed, coaching practitioners perceive these performance indicators as being of the highest value to performance outcomes and scientific research shows these are major discriminatory variables in achieving success particularly at elite standards of play (3). As such, a plethora of research, synthesised in a systematic review (2), has examined goal scoring patterns in elite football. Although there are relatively few published scientific reports that have examined how football play has evolved over time and notably regarding trends in how goals are scored across the same tournaments, goal scoring patterns across multiple FIFA World Cups^TM^ have received some attention in the literature (4–6). However, to our knowledge, no scientific study has investigated goal scoring patterns during the most recent World Cup held in Qatar in 2022. Comparison of findings with those observed in previous tournaments is important especially when accounting for the major technical and tactical evolutions that have recently occurred in the elite games (7).

Soccer teams at elite standards frequently use a range of key performance indicators (KPIs) to measure tactical and technical performance underpinning goal scoring actions. Analyses during World Cup matches have provided pertinent information relating to the actions preceding goals scored (e.g., set pieces or open-play situations), the game period (e.g., final 15-min) and pitch zones (e.g., penalty area) in which goals were scored, the body part used (head, left vs. right foot), and the final action before the goal (e.g., pass, cross) (6). For example, analyses of international play showed that the majority of goals scored (∼70%) were from open play actions (8) and more precisely, 60% from a positional attack, 20% during a direct attack and 20% during a counterattack. While set-pieces generally represent around 30% of goals scored (4, 9–11) a record number of goals scored from set-play situations was observed during the 2018 FIFA World Cup held in Russia. The period of the match also has a significant influence on goal scoring patterns (4, 12, 13). For example, most goals are scored between the 76th and the 90th min with a significant number scored during additional time (6). This trend has been linked with the accumulation of fatigue represented by a decrease in physical performance towards the end of play (14). A temporal analysis of goals scored in the last World Cup is of particular interest especially when accounting for the recent rule changes regarding the number of substitutes allowed and the increased amount of added time.

Recently, these simple event statistics gathered from coding games have evolved into higher value metrics such as Expected Goals (xG) and Expected Goals On-target (xGOT). xG values quantitatively determine the quality of a scoring chance and how likely it is that it will be scored using a combination of variables prior to, and up to, the exact moment the shot was taken. These variables include the distance and angle the shooter was in relation to the goal, positions and pressure of defenders and goalkeeper, the body part with which the shot was taken, and type of assist or previous action (e.g., cross, set-piece, dribble, …). xGOT measures the likelihood of an on-target shot resulting in a goal based on the combination of the underlying chance quality (xG) and the end location of the shot within the goalmouth (15). Machine-learning techniques on large datasets of scoring chances across multiple teams and competitions have been used to develop these metrics which are shown to be key and discriminating indicators of scoring performance in elite soccer match-play (16, 17). However, no scientific study has to our knowledge, published data on these key advanced metrics during a World Cup tournament or compared trends in these across recent World Cups.

The aims of this study were twofold: (1) to determine patterns of goals scored during the 2022 FIFA Qatar World Cup, and (2) compare these patterns with data from the 2018 FIFA World Cup held in Russia.

## Methods

### Sample

Goal scoring patterns were analysed and compared between the most two recent FIFA World Cups (WC): Russia 2018 (WC 2018) and Qatar 2022 (WC 2022). A total of 341 goals were scored across the two competitions: (goals scored in penalty shootouts were not considered): 169 goals during WC 2018 and 172 goals during WC 2022). All performance data and video were extracted with permission from the ProVision match analysis software (StatsPerform Ltd, Chicago USA). Match event data available in the software was coded internally by trained company performance analysts. As this study used secondary data which can be freely collected, ethics committee approval was not considered necessary.

### Variables

For this study, qualitative and quantitative dependent data were analysed separately. Table 1 provides information on the different variables analysed for all goals scored. These included the period in which goals were scored, the type of action and different types of play, body part used, defensive pressure and possession duration. For all these dependent variables, a sub-analysis was performed to examine goalscoring patterns specifically during the group and the knockout phase. Finally, the values for expected goals (xG) (16) are provided. xG measures the quality of a chance by calculating the likelihood that it will be scored on a scale between zero and one, where zero represents a chance that is impossible to score, and one represents a chance that a player would be expected to score every single time. Similarly, values are provided for expected goals on-target defined as the measure of the likelihood of an on-target shot resulting in a goal, based on the combination of the underlying chance quality (xG) and the end location of the shot in the goal (17).

**Table 1:** Description of the variables in the study and their (sub)categories.^1^

Type of data	Variable	Description
Qualitative data	Time period of goal scored	9 match periods: - P1: 0–15 - P2: 16–30 - P3: 31–45 - P4: first-half added-time - P5: 45–60 - P6: 61–75 - P7: 76–90 - P8: second-half added-time - P9: Extra-time
	Type of Action	Open Play, Set Piece, Own Goal
	Type of play	OP Positional Attack (PA), OP Fastbreak (FB), Penalty kick (PK), Direct Free Kick (FK), Indirect Free Kick (IFK), Corner Kick (CK), Throw-In (TI), Own Goal (OG).
	Body part	Right foot (RF), Left foot (LF), head (H), Own Goal (OG).
	Defensive pressure	Low, Moderate, High.
Quantitative data	Possession duration (only for goals scored in Open Play)	
	xG	
	xGot	
	Shooting Goals Added (SGA)	(xGot-xG)

For the analysis of *Defensive Pressure*, *xG* mean and *xGot* variables, own goals scored were not considered as data for these three variables were not available. For *xG*, *xGot* and *Shooting Goals Added (SGA)* values the analysis was performed in two steps: the first being a general analysis of all goals scored and the second, a separate analysis for each type of action: Open Play and Set Pieces.

### Reliability

To evaluate the reliability of the data utilised, we performed an inter-operator agreement. The first author performed an analysis of 171 goals (50% of the total sample). He is an UEFA B licensed and Performance Analyst in an Elite French Women's Club Academy and has a master's degree in Sport Coaching and a PhD in Sports Sciences and thus can be considered an expert observer in football. Data generated from analysis was compared with that extracted from the ProVision match analysis software coded by company analysts (StatsPerform Ltd, Chicago USA). Cohen's Kappa was applied to compare datasets. Overall, we found excellent reliability with a coefficient of 0.90 for *Type of play* and *Type of action*, 0.97 for *Body part* and 0.93 for *Defensive pressure.* Reliability for *xG* and *xGot* could not be determined as these metrics were calculated by StatsPerform using its propriety algorithms.

### Data analysis

Descriptive statistics were used to determine frequencies and percentages for the different variables. A Chi-square test was performed to assess differences between competitions for all qualitative variables except for xG and xGot for which only cumulative values are presented. Statistical analysis was performed using IBM SPSS Statistics (Version 23, SPSS, Inc., Chicago, IL). The Kolmogorov-Smirnov test was used to determine the normality of the variables for *Possession Duration* across both tournaments. Data demonstrated a non-normal distribution. As such, mean values for these variables were compared using a nonparametric Mann–Whitney *U*-test for independent samples. The level of significance was established at.05. For effect size measures, Cramer V values were calculated. ES were interpreted as small (0.1–0.3), moderate (0.3–0.5), and strong (>0.5) (18).

## Results

### Game period

**Figure 1:**
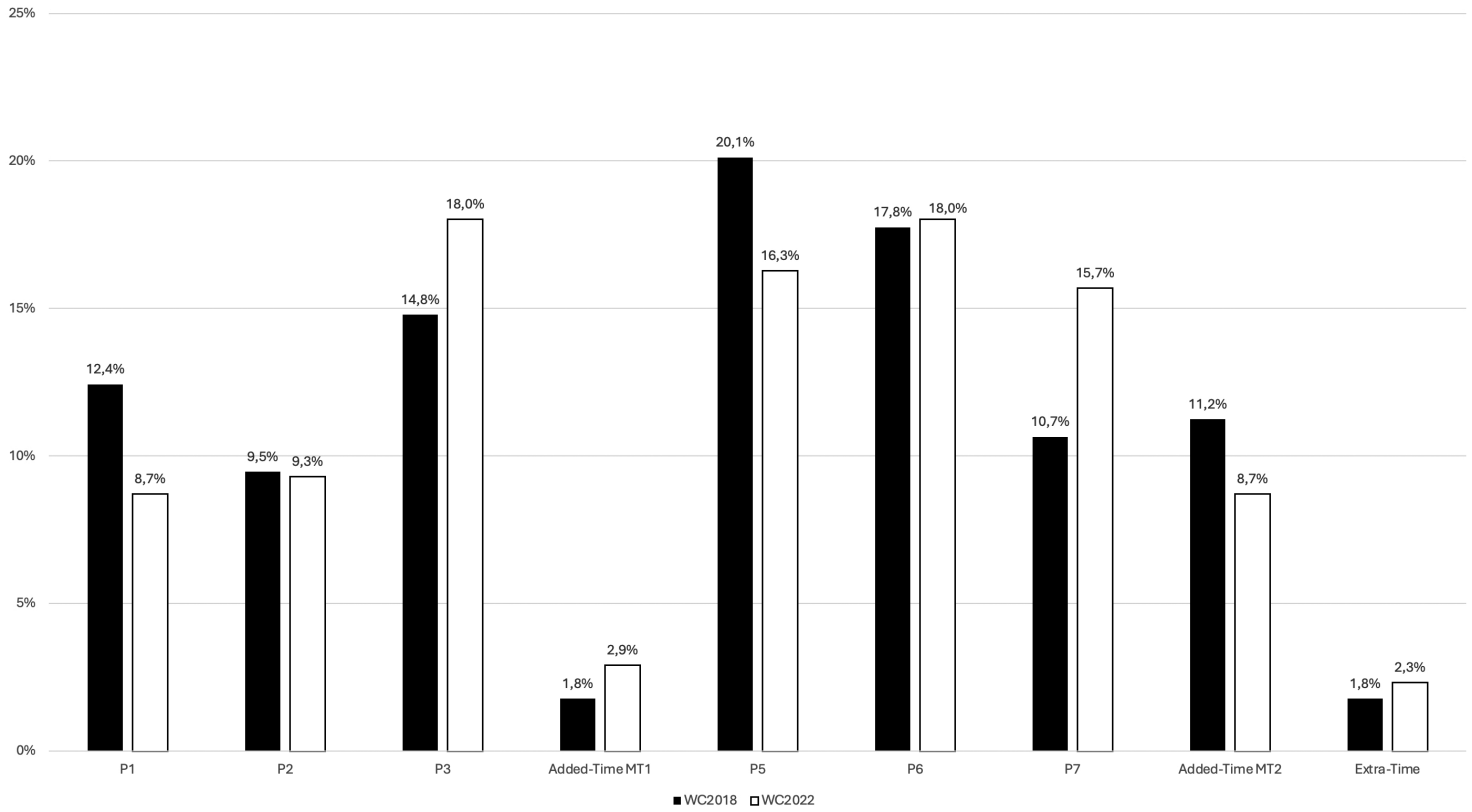
Time period in which goals were scored in the 2018 and the 2022 World Cups. Data is presented as a percentage of total goals scored per period for each competition (CW2018: *N* = 169; CW2022: *N* = 172).

The mean number of goals scored in each match period across the two competitions is reported in Figure 1. No significant differences were observed (*χ*^2^ = 5.127; *p* = 0.744). In the sub-analysis, no significant differences were observed across tournaments for the Group and Playoff phases. The majority of goals was scored during the second-half of play (P5, P6, P7, P8): 59.8% for WC2018 and 58.7% for WC2022. A trend showing a progressive decrease in goals scored across the second half can be observed although the proportion of the total goals scored was highest during the final 15-min plus added time.

### Type of action

A significant difference (*χ*^2^ = 17.953; *p* = 0.000; ES = 0.23) in frequencies for the different types of action preceding goals scored was observed between the two World Cups. The number of Own Goals decreased 6-fold in WC 2022 (12 OG in 2018 vs. 2). In Figure 1, a decrease in the proportion of the total goals scored from set pieces can be observed (38.5% in 2018 vs. 24.4% in 2022). The observation of adjusted residuals revealed that all these differences were significant.

**Figure 2:**
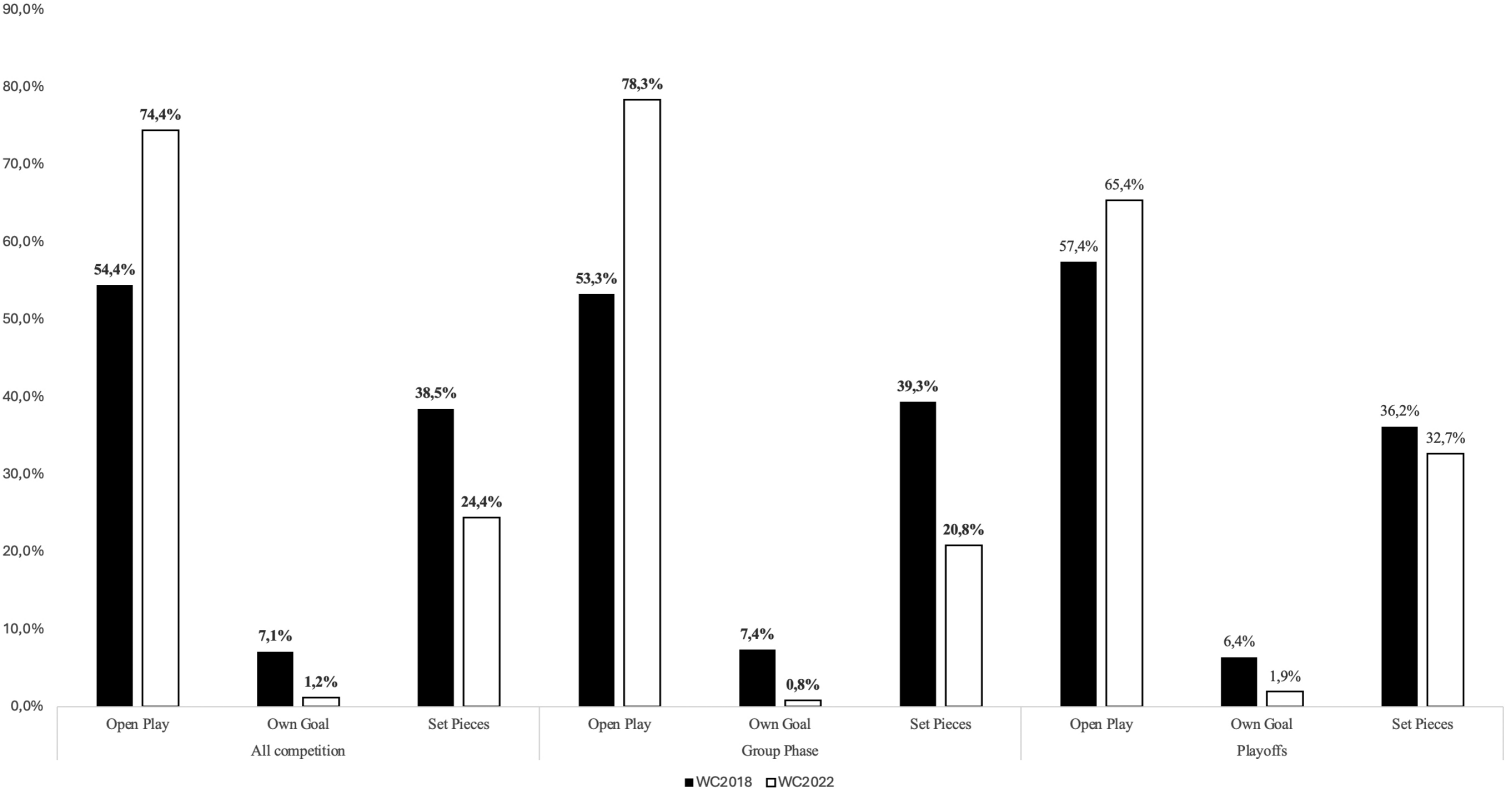
Percentage of total goals scored (CW2018: *N* = 169 and CW2022: *N* = 172) according to the type of action and tournament phase in the 2018 and the 2022 World Cups.

The sub-analysis demonstrated (Figure 2) a significant difference between tournaments for the types of goal scored in the Group Phase (*χ*^2^ = 18.921; *p* < 0.001; ES = 0.28) but not in the Playoffs (*χ*^2^ = 1.555; *p* = 0.46; ES = 0.13). The observation of adjusted residuals revealed that all differences in the Group Phase were significant. A decrease in the number of Own Goals and Goals scored specifically from set pieces during the group phase (respectively −6.6% and −18.5%) can be observed leading to a concurrent increase in the number of goals scored from Open Play situations (+25%).

### Type of play

A significant difference was observed for the number of goals scored during the various types of play between the two competitions (*χ*^2^ = 22.031; *p* = 0.003; ES = 0.25) (Table 2). This result was due to an increase in the frequency of goals scored from *Open Play* actions (positional attack & direct attack) between 2018 and 2022 (+21.3%) and a decrease in the number of *Own Goals* (12 OG in 2018 vs. only 2 in 2022).

**Table 2:** Comparison of goals scored according to the type of action and tournament phase between the 2018 and 2022 FIFA world cups.

Competition	Type of action	Set pieces					Open play		Own goal	Total
		CK	FK	IFK	PK	TI	PA	FB	OG	
WC 2018	*n*	23	7	12	22	1	79	13	12	169
	%	13.6	4.1	7.1	13	0.6	46.7	7.7	7.1	100
	Adjusted Residuals	1.8	1.7	.5	.9	1.0	−**4**	.5	**2**.**8**	** **
WC 2022	*n*	13	2	10	17	0	117	11	2	172
	%	7.6	1.2	5.8	9.9	0	68	6.4	1.2	100
	Adjusted Residuals	−1.8	−1.7	−.5	−.9	−1.0	**4**	−.5	**−2**.**8**	
Total	*n*	36	9	22	39	1	196	24	14	341
	%	10.6	2.6	6.5	11.4	0.3	57.5	7	4.1	100
Group phase										
Competition		Set pieces					Open play		Own goal	Total
		CK	FK	IFK	PK	TI	PA	FB	OG	
WC 2018	*n*	16	6	8	18	0	55	10	9	122
	%	13.1	4.9	6.6	14.8	0	45.1	8.2	7.4	100
	Adjusted Residuals	1.4	1.4	.8	1.8		**−4.3**	.7	**2**.**6**	
WC 2022	*n*	9	2	5	9	0	87	7	1	120
	%	7.5	1.7	4.2	7.5	0	72.5	5.8	0.8	100
	Adjusted Residuals	−1.4	−1.4	−.8	−1.8		**4.3**	−.7	**−2**.**6**	
Total	*n*	25	8	13	27	0	142	17	10	242
	%	10.3	3.3	5.4	11.2	0	58.7	7	4.1	100
Playoffs										
Competition		Set pieces					Open play		Own goal	Total
		CK	FK	IFK	PK	TI	PA	FB	OG	
WC 2018	*n*	7	1	4	4	1	24	3	3	47
	%	14.9	2.1	8.5	8.5	2.1	51.1	6.4	6.4	100
	Adjusted Residuals	1.1	1.1	−.2	−1.0	1.1	−.7	−.3	1.1	
WC 2022	*n*	4	0	5	8	0	30	4	1	52
	%	7.7	0.0	9.6	15.4	0.0	57.7	7.7	1.9	100
	Adjusted Residuals	−1.1	−1.1	−.2	1.0	−1.1	.7	.3	−1.1	
Total	*n*	11	1	9	12	1	54	7	4	99
	%	11.1	1.0	9.1	12.1	1.0	54.5	7.1	4.0	100

PA, OP positional attack; FB, fastbreak, PK, penalty kick; FK, direct free kick; IFK; indirect free kick; CK, corner kick, TI, throw-in; OG, own goal.

Bold values are significant values.

The sub-analysis across tournament phase demonstrated significant differences for the group (*χ*^2^ = 21.778; *p* = 0.001; ES = 0.30) (Table 2) but not for the playoff phase (*χ*^2^ = 5.835; *p* = 0.56; ES = 0.24) (Table 2). Indeed, in Table 3, a significant increase in the number of goals scored from OP Positional Attack during the group phase in 2022 (72.5% in 2022 against 45.1% in 2018) and, conversely, a significant decrease of the number of Own Goals in 2022 (0.8% in 2022 against 7.4% in 2018) can be observed.

**Table 3:** Xg and xGot cumulated values compared with total goals scored in the 2018 and the 2022 FIFA world cups.

Type of actions	Competition	N	xG	Diff NGoals vs. xG	xGot	Diff NGoals vs. xGot	Shooting Goals Added (xGot – xG)
All goals	WC2018	157	171.7	−14.7	163.9	−6.9	−7.8
	WC2022	170	175.2	−5.2	176.8	−6.8	+1.6
Open play	WC2018	92	105.5	−13.5	102.6	−10.6	−2.9
	WC2022	128	122.8	+5.2	131.5	−3.5	+8.7
Set pieces	WC2018	65	66.1	−1.1	61.3	+3.7	−4.8
	WC2022	42	52.4	−10.4	45.3	−3.3	−7.1

### Body part

A significant difference for the frequency of goals scored according to the body part used by players was observed between the 2018 and the 2022 World Cups (CW2018: *N* = 169; CW2022: *N* = 172, *χ*^2^ = 8.646; *p* = 0.03; ES = 0.15). Close to 50% of the total goals were scored using the right foot. We observed a significant decrease in the number of Own Goals in 2022 (7.1% in 2018 against 1.2% in 2022).

### Defensive pressure

A decrease in goals scored in low pressure contexts (−11.7%) and an increase in goals scored in moderate and high-pressure contexts (respectively +7.7% and +4.0%) was reported between the two competitions although these differences were not statistically significant (*χ*^2^ = 4.778; *p* = 0.09).

### Possession duration

No difference in the mean possession duration prior to goals scored was observed between the two World Cups (*p* = 0.211). In 2018, the mean duration was 25.9 s (SD = 23.7) vs. 33.6 s (SD = 34.46) in 2022.

### xG and xGot

Table 3 presents the cumulated values for *xG* and *xGot* overall and according to the type of action compared with the number of goals scored respectively across the two competitions. For *xG*, we can observe that in the category “All goals”, teams scored less goals than expected in both World Cups (respectively −14.7 and −5.2 for 2018 and 2022). A similar trend was observed for set-plays and open-play situations across both tournaments although in 2022 more goals than expected were scored from open-play (+5.2).

The comparison (or difference) between xGot and xG (xGot-xG), or Shooting Goals Added (“SGA”) to compare the quality of the scoring opportunity and the quality of the shot, presents values of −7.8 in 2018 vs. +1.6 in 2022 for the category “All goals”. In the category “Open Play”, SGA was positive in 2022 (+8.7) and negative in 2018 (−2.9). SGA values were negative for both competitions in the category “Set Pieces”.

## Discussion

The aim of the present study was to analyse and compare goal scoring patterns between the two most recent two FIFA World Cups (WC): Russia 2018 and Qatar 2022. Main findings were the significant differences observed across the two competitions for the type of action, type of play and body part used by players prior to goals scored whereas no differences were reported for the temporal periods in which the goals were scored. In both tournaments, teams underperformed regarding goals scored in relation to xG values.

### Game period

The temporal analysis of goals scored showed no significant differences between WC 2018 and 2022. In both tournaments the majority of goals was scored during the second half (respectively 59.8% for WC 2018 and 58.7%). Further analysis showed that the most prolific period for goal scoring in both competitions was between the 75th min and the end of regular time. This result corroborates data reported by Kubayi and Toriola (5) and Mićović et al. (6) from previous WC tournaments showing that most goals were scored towards the end of matches. It has been suggested that the increase in goals scored towards the end of matches is likely linked to an accumulation of physical and mental fatigue (18). It is noteworthy that when the final 15-min period is merged with added-time, a small increase in this temporal trend occurred in 2022 (21.9% in 2018 vs. 24.4% in 2022). This observation suggests that the new rule permitting the use of 5 substitutions in 2022 and hence benefiting from a larger number of “fresher” players on the pitch, did not help teams prevent goals being scored towards the end of games. Conversely, this rule change may potentially have aided teams in scoring more goals towards the end of play. Unfortunately, information regarding the tactical role of substitutes and their individual contributions (technical and physical notably) was unavailable here and arguably merits investigation in future studies. Finally, this result might also be linked to the increase in added time observed during the 2022 WC and again, further research in future international tournaments is warranted to examine trends relating to the time period in which goals are scored.

### Type of action and type of play

A significant decrease in the number of own goals was observed between 2018 and 2022 (respectively 12 and 2). This difference can be explained straightforwardly by the record number of *Own goals* for a WC recorded in 2018. Indeed, Kubayi and Toriola (5), reported lower values, ranging between 2 and 5 OG scored per tournament, between 1998 and 2014.

Similarly, a decrease in the number of goals scored from *Set Piece* situations can be observed in 2022. This result can again be linked to the record number of goals scored from these game situations in Russia 2018 (24.4% vs. 38.5%). Data reported previously shows proportions ranging between 20% and 30% of total goals scored (4, 10, 11, 19). It is difficult to provide a reasonable explanation for this drop in set-piece goals although a link can be made with data reported in a recent article (10) which showed that both the overall number of attacking set-pieces taken and the proportion of these ending in a shot at goal declined considerably in the 2022 tournament.

A concurrent increase in the proportion of goals scored in Open Play (Positional & Direct attacks) occurred between 2018 and 2022 (+21.3%). This difference can be explained by the greater number of goals scored from positional attacks in 2022 vs. 2018 (79 vs. 117). A reasonable explanation for this finding might be linked to the expected goal statistic. At elite standards, teams are now attempting to work the ball into more dangerous areas more often to maximise their attacking phase and attempt to increase the quality of their scoring opportunities rather than shooting from greater distances which substantially decreases the likelihood of scoring (20).

### Defensive pressure, expected goals (xG) and xGOT

No significant evolution regarding *Defensive Pressure* contexts across the two WC was observed although the proportion of goals scored in a low-pressure context decreased in 2022 (−11.7%) while the opposite trend was observed for goals scored in moderate and high-pressure contexts respectively (+7.7% and +4.0%).

The analysis of cumulated expected goals values (xG) or the quality of the chance and likelihood of it becoming a goal, shows that in both World Cups, the teams overall scored fewer total goals than expected although the total goals-Xg difference was lower in 2022 (2018 = −14.7, 2022 = −5.2). This result suggests that teams generally lacked efficiency when converting their chances in relation to the quality of these scoring opportunities. This was particularly true in 2018 where analysis of Shooting Goals Added (“SGA”) values (comparing the quality of the scoring opportunities [xG] and the quality of the shots that were on target [xGot]) reported a negative value of −7.8 suggesting that even when teams managed a shot on target this was frequently low in quality.

Regarding the total goals scored from “Open Play” situations, teams can be considered to have been substantially more efficient in the 2022 World Cup. Indeed, an xG value showing that approximately 123 goals from open play should have been scored compared to an actual figure of 128 goals. This observation is supported by the increase (albeit non-significant) in the number of goals scored in high-pressure defensive contexts suggesting greater efficiency. It is noteworthy that the xGot value, which demonstrates the quality of on-target shots, was higher than the total number of goals scored and notably in comparison with the cumulated xG value. Teams therefore frequently produced more high-quality on-target shots in 2022 but these did not lead to more goals being scored which suggests that the goalkeepers performed well when preventing goals (1).

Finally, as mentioned earlier, a record number of goals scored from set-piece situations was scored during the 2018 WC. While this finding could simply be linked to the lower number of set pieces taken in 2022 (20), it might also be explained by the better cumulated xG vs. actual total goal values. Indeed, in 2022, the quality of the set-piece opportunities should have led to approximately 52 goals being scored whereas only 42 goals in total were in fact scored. In contrast, the difference observed in 2018 was considerably smaller: xG=∼66 vs. 65 goals scored thereby demonstrating greater efficiency than in 2022.

### Limitations and perspectives

Here, the authors utilised information obtained from a commercial soccer match performance data provider. Hence, this limited the study's scope to the variables available and the provider's definitions. However, this choice enabled inclusion of the provider's propriety advanced expected goal and expected goal on-target statistics which to our knowledge have not yet been compared in the scientific literature across WC tournaments.

Future research could investigate whether differences occur across WC in the sequences of play (e.g., number of passes per sequence) and the types of final actions (e.g., passes, cross, dribble) prior to goals scored. A study across the last four World Cups has also highlighted substantial differences in the distance of shots from the goal with a recent tendency for teams to attempt their shots from closer positions (20). This research could be extended to other attacking actions, for example, the pitch zones from which crossing actions were performed.

## Conclusions

The aim of this study was to compare trends in goal scored across the two most recent FIFA World Cup tournaments: Russia 2018 and Qatar 2022. No differences were observed between the two competitions regarding the temporal period in which goals were scored. However, most goals were scored during the second half and more precisely between the 75th min and the end of the regular time confirming trends observed across previous tournaments. This suggests a need, even at the very highest levels of play, for strategies and techniques to counteract fatigue, both physical and mental. In contrast, statistically significant differences across the two tournaments were reported for the body part used, the type of action, and type of play prior to scoring a goal. The latter differences can in part be explained by the record number of own goals and goals scored from set-piece situations observed during the 2018 World Cup whereas goals from open-play situations occurred more frequently in 2022. As patterns varied across the World Cups, the present results suggest that players must acquire technical and tactical skillsets that enable them to respond to the demands and trends in play emerging in individual tournaments. Finally, in both World Cups, teams generally underperformed regarding the number of goal attempts converted into goals when the quality of the scoring opportunities was accounted for. This can be explained by the lower quality of the goal attempts and/or high stopping ability of goalkeepers and suggests that even in international standard football, there is a need to train and optimise players' shooting ability.

## Data Availability

The raw data supporting the conclusions of this article will be made available by the authors, without undue reservation.

## Footnotes

^1^*Variables are defined in StatsPerform’s glossary:* *https://www.statsperform.com/opta-event-definitions/*.
